# Validation of Remote Sensing Retrieval Products using Data from a Wireless Sensor-Based Online Monitoring in Antarctica

**DOI:** 10.3390/s16111938

**Published:** 2016-11-17

**Authors:** Xiuhong Li, Xiao Cheng, Rongjin Yang, Qiang Liu, Yubao Qiu, Jialin Zhang, Erli Cai, Long Zhao

**Affiliations:** 1 College of Global Change and Earth System Science, Beijing Normal University, No. 19, Xin Jie Kou Wai Street, Beijing 100875, China; lixh@bnu.edu.cn (X.L.); xcheng@bnu.edu.cn (X.C.); toliuqiang@bnu.edu.cn (Q.L.); 201221490009@mail.bnu.edu.cn (J.Z.); 201421490024@mail.bnu.edu.cn (E.C.); zhaolong@mail.bnu.edu.cn (L.Z.); 2 Joint Center for Global Change Studies, Beijing 100875, China; 3 State Key Laboratory of Environmental Criteria and Risk Assessment, Chinese Research Academy of Environmental Sciences, No. 8, Da Yang Fang, An Wai, Chao Yang, Beijing 100012, China; 4 Institute of Remote Sensing and Digital Earth of Chinese Academy of Sciences, Beijing 100101, China; qiuyb@radi.ac.cn

**Keywords:** Antarctica, wireless sensor based online monitoring platform, remote sensing product, validation

## Abstract

Of the modern technologies in polar-region monitoring, the remote sensing technology that can instantaneously form large-scale images has become much more important in helping acquire parameters such as the freezing and melting of ice as well as the surface temperature, which can be used in the research of global climate change, Antarctic ice sheet responses, and cap formation and evolution. However, the acquirement of those parameters is impacted remarkably by the climate and satellite transit time which makes it almost impossible to have timely and continuous observation data. In this research, a wireless sensor-based online monitoring platform (WSOOP) for the extreme polar environment is applied to obtain a long-term series of data which is site-specific and continuous in time. Those data are compared and validated with the data from a weather station at Zhongshan Station Antarctica and the result shows an obvious correlation. Then those data are used to validate the remote sensing products of the freezing and melting of ice and the surface temperature and the result also indicated a similar correlation. The experiment in Antarctica has proven that WSOOP is an effective system to validate remotely sensed data in the polar region.

## 1. Introduction

Polar regions are critical in global climate change. Especially as the Antarctic is considered the last piece of land protected from human pollution. However, due to the harsh environment, only a very small fraction of the vast area of the Antarctica is accessible to humans. Only a few regions can support continuous observations and investigations. Most ground-based observations about the polar environment are conducted at or in close proximity to those research stations which had been well-constructed to some extent. Since the 1970s, humans have begun to explore Antarctica with the help of satellite remote sensing. Information from satellites has become the most effective source to acquire geophysical characteristics and parameters about the Antarctic ice sheet, including surface topography and elevation, albedo, temperature, humidity, wind speed and direction, snow grain size, and snow melt. These parameters are significant for the study of the Antarctic ice sheet formation and evolution, as well as response to global changes. However, as the frequency of remote sensing observation is relatively low, the acquired parameters are not continuous in time. Moreover, the satellite-retrieved parameters need to be validated with the ground truth data. The ground-based observations are very difficult to conduct in Antarctica because of the harsh environment: researchers cannot inspect large-scale areas and ordinary equipment cannot work continuously [1,2].

Wireless sensor network technology is a new ground-based remote sensing technology which integrates modern communication, computers, wireless networks, and remote sensing. Compared to the traditional automatic weather station or manual survey, it has a relatively low cost and flexible settings to acquire field observation data [3,4]. By lapping different sensors or sets of sensors properly, the nodes of the wireless sensor network can continuously acquire physical factors such as temperature, humidity, wind speed, wind destination, snow depth, global position system (GPS) location, and atmospheric pressure [5,6,7,8]. This new technology has the potential to make up for the gap of sparse observation stations in regions of harsh environment and also support the retrieval, validation, and assimilation of satellite remote sensing data [5,9]. However, because of the hard accessibility and unfriendly environment, especially the extremely low temperature and insufficient solar energy, the successful application of wireless sensor networks in the Polar Regions are seldom reported [10]. The SEAMONSTER Sensor Web Research Team of the University of Alaska Southeast (Sitka, AK, USA) observed the Lemon Iceberg near Juneau, and modeled the process of its melting in response to climate changes [7]. Pirazzini and Broeke used observational data of albedo at an automatic weather station as a reference to assess the quality of generated products for albedo. In the Antarctic, [11,12,13] installed a prototype system of a wireless sensor network in the Antarctic Ice Dome A, which is produced by Crossbow Technology, Inc., a leading US company in Intelligent Sensors and has the basic observing function of meteorological parameters [14]. Currently, most monitoring platforms in polar regions are aimed at a single research objective and collect relatively few parameters. The transmission of different forms of information and the construction of a comprehensive observation network to support remote sensing applications are an urgent requirement in the polar regions, especially in Antarctica.

Temperature change is one of the focuses of the Antarctic Research in recent years. Hugues et al. (2012) studied the Antarctic temperature changes during the last millennium [15]. Marshall et al. (2011) analyzed the regional change of temperature relationship in Antarctica [16]. Bian et al. (2012) analyzed the vertical structure and seasonal change of atmosphere temperature at Zhongshan Station [17]. Xin et al. (2014) simulated and evaluated the 2-m temperature over Antarctica [18]. Wang and Hou (2011) analyzed the spatial distribution of 10 m firn temperature in an Antarctic ice sheet [10]. Liu et al. (2015) constructed an effective method for Antarctic ice surface temperature retrieval [9]. 

In this study, we developed a multi-purpose wireless sensor-based online monitoring platform and applied it to the extreme environments in Antarctica to validate remote sensing products.

## 2. The Wireless Sensor-Based Online Monitoring Platform (WSOOP) for Extreme Environments

### 2.1. The Architecture of WSOOP

**Figure 1:**
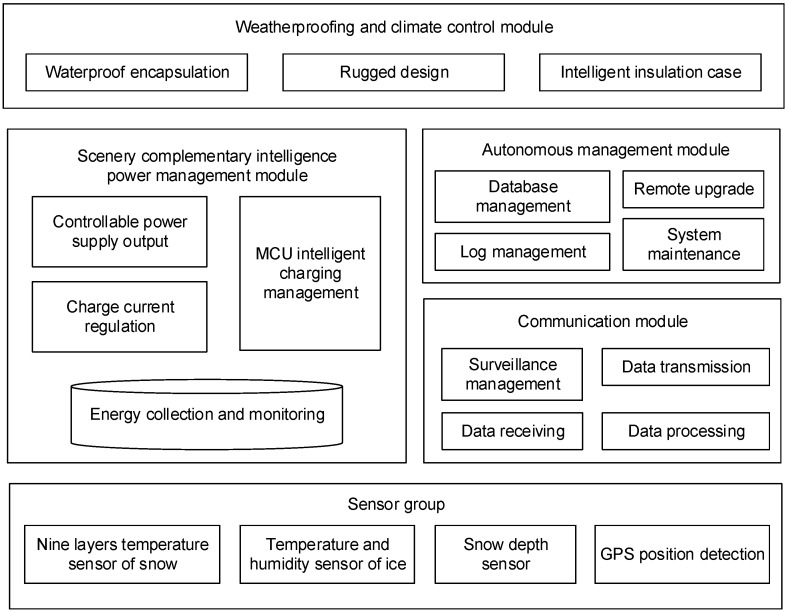
The architecture of the WSOOP.

The wireless sensor-based online monitoring platform for extreme environments is designed to work in the polar region; thus, its devices particularly require cold resistance. Additionally, because of the difficulty for researchers to access those observation sites, the equipment needs to have strong self-management ability. Moreover, the harsh environment in polar regions demands more in terms of the equipment’s energy management and control than ordinary conditions. According to the above demands, the architecture of WSOOP is divided into five modules: the signal detection module, the self-management module, the communication module, the energy management module and the module of circuits and packaging against harsh environments as shown in Figure 1.

### 2.2. The Special Design against Extreme Environments

#### (1) On cold resistance and low power consumption

Due to the harsh environment and the low temperature in which the platform is supposed to be installed, ordinary electronic components cannot work regularly. Therefore, the system entirely adopts industrial components and chips that can work under temperatures as low as −40 °C. As a specially produced temperature-compensation crystal oscillator with cold resistance, the matched crystal oscillator can still function at −40 °C (see Table 1).

**Figure 2:**
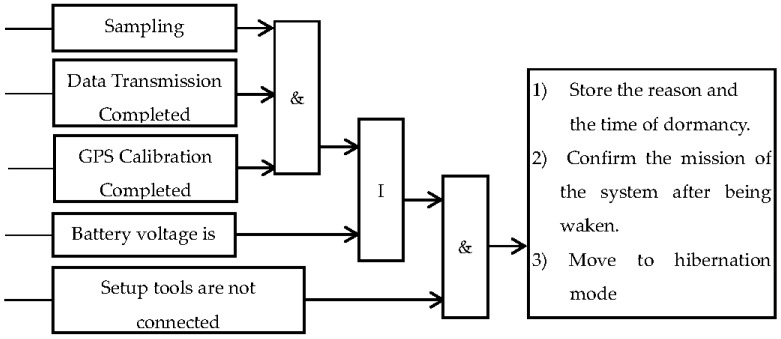
Low power consumption and the logic of management and control in the ZKOS.

The designed temperature-compensation circuits can guarantee both regular function and accuracy of the components including the crystal oscillator, the amplifier, etc. Moreover, the platform is equipped with a hypothermic Real-Time Control (RTC) module, which has inner crystal oscillators. This allows for the platform to provide timing of high accuracy and low error. Figure 2 shows the sleeping strategy in low power consumption and the logic of management and control in the ZKOS (ZhongKe Operation System) [14].

#### (2) On integrated multi-interfaces

**Figure 3:**
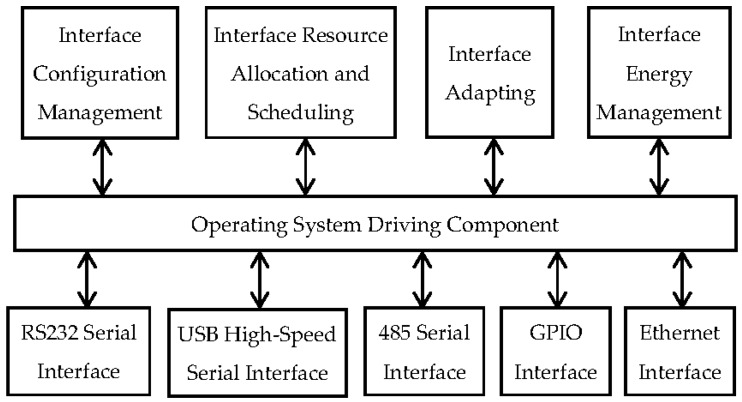
The integrated module of ports with multiple functions.

Scientific research often requires synchronous measuring data of multiple parameters, so one set of equipment has to be linked with several different sensors or devices from different producers (even different countries). To improve its universality and extensibility, an integrated module of ports with multiple functions is designed for signal collection and control, which is shown in Figure 3.

The WSOOP is designed with the core board separated from the collecting board. On the core board, ports, USB, and Ethernet ports, which can be used with most of the current sensors, are integrated. The collecting board is linked with all of the sensors. For digital sensors, it provides communication interfaces to the processors. For analog sensors, if the signals from the sensors are needed to be magnified and transferred, it will provide respective function components and will link the final signal to the processors. For pulse-output sensors, it transfers the signals from the sensors and links them to the processors.

#### (3) On embedded smart insulated case

Because the lowest temperature where most of the platform components and modules can work is −40 °C, a small number of them cannot work in temperatures below −20 °C, and thus they cannot work in the cold environment of the Antarctica. Therefore, an embedded smart insulated case was developed. Moreover, it can maintain different levels of temperatures with several parameters in the respective modified programs. The case has two tiers and two layers of PTC (Positive Temperature Coefficient—a special ceramic material that can send out heat of constant temperature). The outer tier is designed for the equipment of which the extreme working temperature is −40 °C, whereas the inner tier is designed for the equipment with an extreme working temperature of −20 °C. Both the tiers of equipment are filled with insulating material to help maintain their own heat. Additionally, the temperature inside the equipment is tested. Once it falls below the extreme temperatures, the equipment is duly heated.

#### (4) On wind-solar complementary smart system of power management

The wind-solar complementary smart system of power management is developed based on ZKOS. With this system, researchers can achieve remote control, including starting, turning off, and restarting the power system (time points, time periods, and modes of the power starting and ending can all be programmable), outputting a direct-current power of 12 V, 5 V, and 3.3 V, etc., protecting the system when an overcharge or over-discharge occurs (it acquires the voltage and decides to close either the system or the charging device). With the support of the wind-solar complementary smart system, the system has been maintained and has operated for more than two years (2012 and 2013). By measuring the voltage on the two sides of the solar panel, it can acquire an accurate time of polar days and polar nights in the Antarctica [14]. 

## 3. The Application of the Platform

### 3.1. The Melting Monitoring System Using the WSOOP 

Except for some blue ice and rocks, ice sheets in the Antarctica are mostly covered by snow. Approximately 9 to 12 percent of the Antarctica surface experiences melting every year [19], which is twice as much as that in Greenland near the Arctic region. Surface melting mainly occurs in December, January, and February, and peaks usually in January. A slight change in atmospheric temperature can bring changes in snow surface humidity on a large scale, and then melt water permeates to the bottom of the ice sheet, which can accelerate ice movement and the collapse of ice shelves. The start time and duration of the annual melting in the Antarctic ice sheet plays an important role in the climate monitoring, so it is essential for global climate change researchers to measure the duration, range, and distribution of polar ice melting. The exact time of melt onset is associated not only with near-freezing air temperatures, but also with the temperature profile below the snow/ice surface.

Currently, the most effective way to quickly explore the states of surface freezing and melting is satellite-based microwave remote technology. Microwave signals are highly sensitive to changes in snow’s dielectric constant, which are sensitive to snow moisture content on ice sheets. The dielectric constant has a significant physical relationship with snow parameters, including snow shape, grain size, density, temperature, humidity, and hardness. Therefore, microwave remote sensing can effectively determine ice sheet freezing and melting status by the inversion of the model with physical mechanism [20,21,22]. 

Till now, there are several algorithms have been developed to detect the time of melting onset in the high latitude regions [23,24,25,26]. To support the validation, as well as algorithm improvements, of remote sensing products, WSOOP was equipped with temperature sensors of high accuracy laid in nine tiers under ice and snow surfaces to monitor the internal temperature profile, together with air temperature and humidity sensors to monitor the environmental parameters. Data were collected every 10 minutes and sent to Beijing every four hours to fit the satellite passing time. With the WSOOP data, the exact melting time of the ice sheet surface can be monitored with high precision.

### 3.2. Deployment of the Wireless Sensor Network

**Figure 4:**
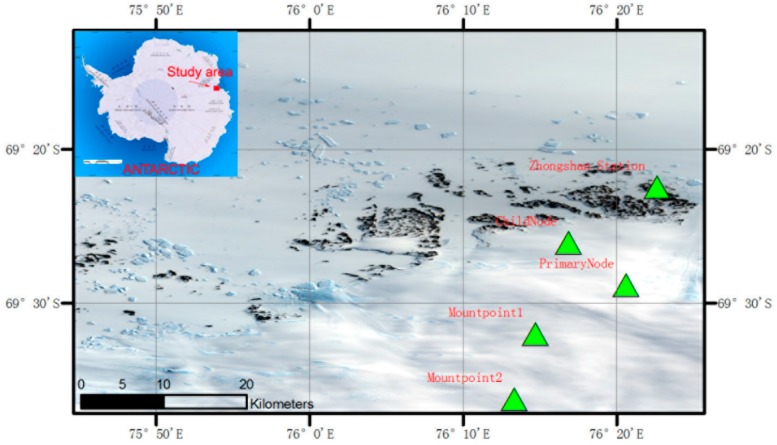
Field installation location of the wireless sensor network devices in Antarctica (the child node, major node, installing point 1 and 2 from the edge of the ice sheet to the left are equipped with observing devices for testing freezing and melting).

**Figure 5:**
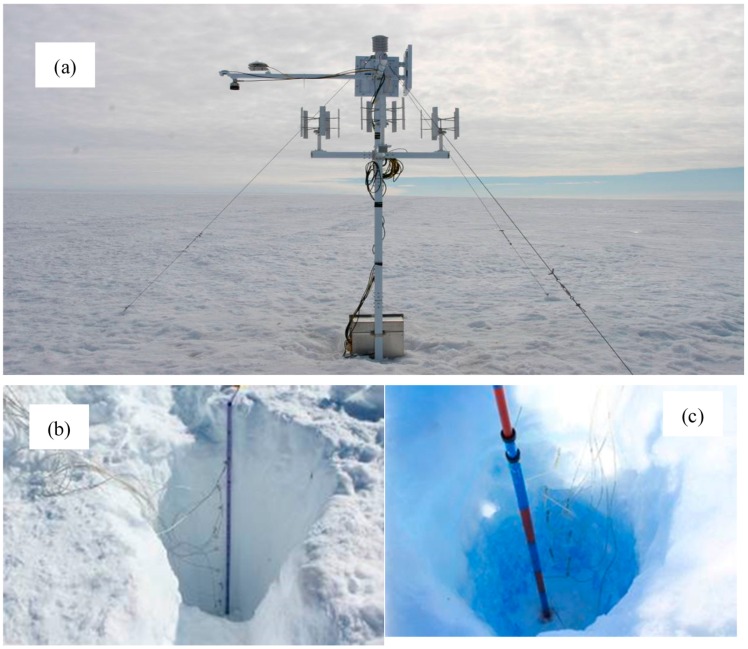
Field installation of the wireless sensor network observing platform ((**a**) the overall effect of the model machine field installation; (**b**) placing of the nine tiers of snow temperature sensors; (**c**) a temperature sensor is placed every 10 cm under the snow surface).

Freezing and melting mainly occurred near the edge of ice sheets in Antarctica and decreased as the elevation increased [27]. Satellite remote sensing was applied to ice sheet freezing and melting monitoring mainly through scatterometers and radiometers—freezing and melting are monitored according to sudden changes in backward scattering and microwave brightness temperature. Microwave brightness temperature is not completely consistent with freezing and melting, and the degree and sequence of freezing and melting vary at different depths of snow. Therefore, according to the monitoring demand of satellite remote sensing, we designed a plan for ground monitoring of freezing and melting on the surface of ice sheets. Four devices are distributed on ice sheets of different altitudes (Figure 4), and nine tiers of snow depth-temperature sensors as well as ice temperature-humidity sensors are set on every point (Table 2, Figure 5). The sensors worked with satellite microwave scatterometers and radiometers, which monitored freezing and melting of ice sheets and tested the accuracy of satellite remote sensing monitoring.

## 4. Data Analysis

The team participated in the 27th and 28th Antarctica Scientific Investigation in China at the end of 2010 and 2011 respectively, conducting the field installation and testing of the system in Antarctica twice. During the 27th scientific investigation, they installed two sets of climate monitoring equipment, which were located on the “major node” and “child node” in Figure 4. The measured parameters included air temperature, humidity and pressure. During the 28th investigation, two sets of equipment for freezing-melting monitoring were installed, which were located at Installation Point 1 and Installation Point 2, respectively, in Figure 4. The measured parameters included air temperature, humidity and nine tiers of snow temperatures. These two sets of equipment have worked steadily until now and have acquired continuous data of freezing-melting monitoring; the analysis below is based on these data.

**Figure 6:**
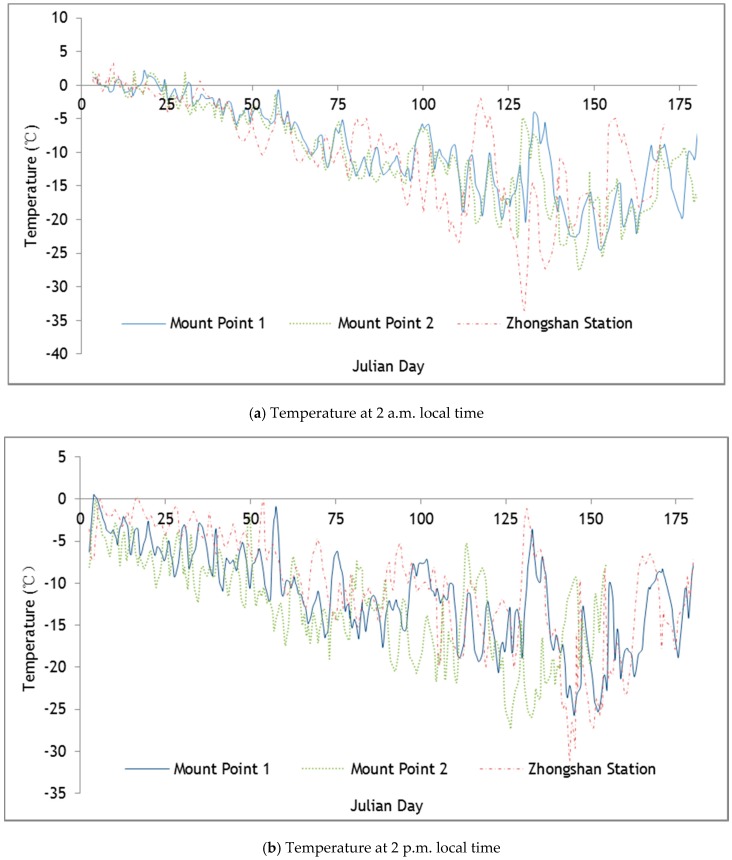
Comparison of the temperature from installation points 1 and 2 and from Zhongshan Station.

**Figure 7:**
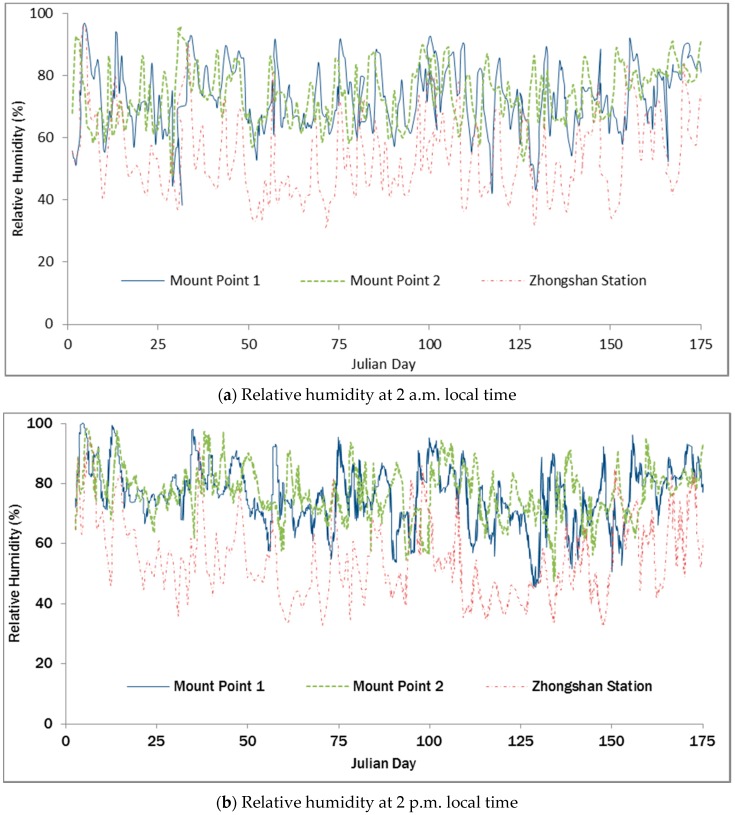
Comparison of the air humidity from installation points 1 and 2 and from Zhongshan Station.

To validate the accuracy of the data, we compared the hourly climate data acquired at Zhongshan Station Antarctica (69°22′24′′ S, 76°22′40′′ E, average altitude of 11 m) with the data acquired by the wireless sensor network devices on “Installation Point 2” (69°36′11.45′′ S, 76°13′22.75′′ E, altitude of 526 m), and then they assessed the reliability of the platform’s data acquisition (Figure 6 and Figure 7). Figure 7 shows the time series of daily temperatures between 2 a.m. and 2 p.m. from January 2012 to June 2012, indicating that the air temperature data from the wireless sensor network device had obvious relevance to the data from Zhongshan Station Antarctica. However, Installation Point 1 and Installation Point 2 are located inland. The temperatures at the two points were similar and lower than that at Zhongshan Station Antarctica. Generally, the temperature decreased from January to June. Since January occurs in the Southern Hemisphere summer, the temperature during daytime could reach slightly above 0 °C. The temperature difference between day and night was obvious. In June, during winter, the temperature decreased to between −10 °C to −30 °C, and the temperature difference between day and night was not obvious. Figure 7 shows the time series of daily air humidity between 2 a.m. and 2 p.m. from January 2012 to June 2012. Compared with the temperature data, the air humidity data from Installation Point 1 and Installation Point 2 were not obviously different from the data of Zhongshan Station Antarctica, but they generally showed higher relative humidity. The analysis above indicates that the meteorological elements acquired by the wireless sensor network for extreme environments are reliable.

## 5. Comparison with Remote Sensing Products

### 5.1. MODIS Surface Temperature Products

**Figure 8:**
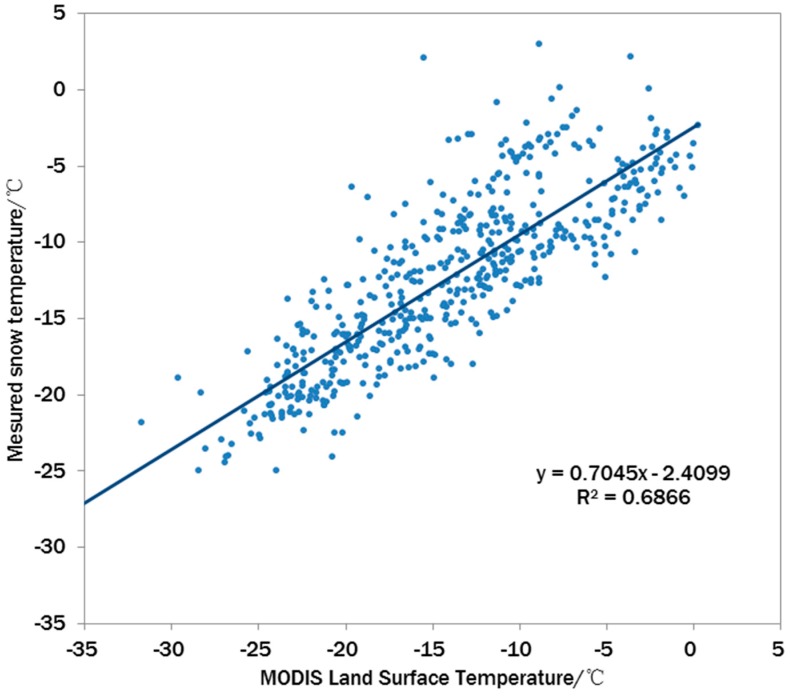
Comparison of MODIS surface temperature products and surface snow temperature data from ground observations.

The MODIS (Moderate Resolution Imaging Spectroradiometer) sensor in the EOS (Earth Observation System) Satellite Program provides global remote sensing observation data in 36 spectral channel from visible to thermal infrared band [28]. Based on the MODIS data, Land Surface Temperature (LST) products (MOD/MYD11A1) with one-kilometer resolution are generated and widely used in meteorology, hydrology, and ecosystem researches. There are numerous studies validating the accuracy of MODIS LST product [29,30]. However, due to the limit of ground observation devices, LST measurement data are hardly available on the permanent ice sheet surfaces in the polar regions. Based on the snow surface temperature observation data at Installation Point 1 and Installation Point 2 nodes of WSOOP, a simple comparison is made between the MODIS LST products and the snow surface temperature. The result of the comparison of all the valid data is shown in Figure 8. In general, the scattered points were distributed around the line of *y* = *x*, which demonstrates the basic accuracy of the surface temperature products. However, the slope of the fitted line of the scattered points was less than 1, indicating that the MODIS surface temperature products slightly overestimated the true snow temperature changes, showing an R^2^ of 0.6866. As the surface temperature in remote sensing is the skin temperature under thermal equilibrium conditions, the changes in skin temperature are larger than those of inner temperature. To a certain degree, this can explain why the slope was less than 1. Moreover, the MODIS product corresponds to pixels of one-kilometer resolution, whereas the footprint of a single node of wireless sensor network can only represent an area of about one square meter. The scale mismatch is also a source of uncertainty in this simple comparison. Thus, the results here are only a demonstration rather than a rigorous validation of the MODIS LST products. 

### 5.2. Microwave Brightness Temperature

**Figure 9:**
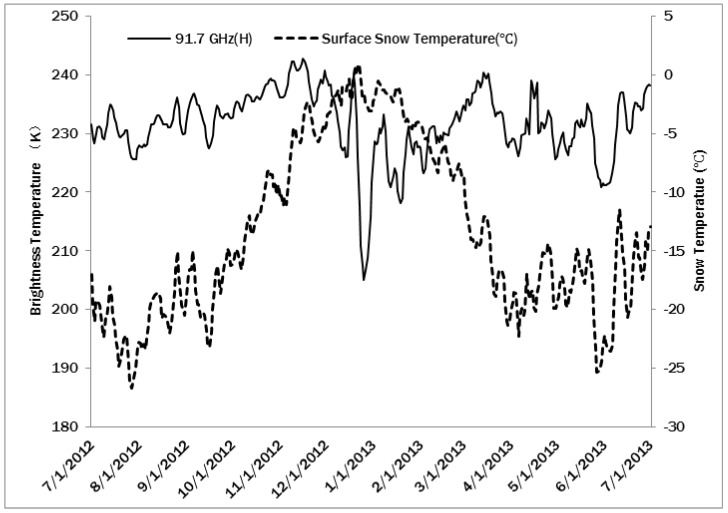
Comparison of the surface snow temperature (five days moving average value) in the daytime at Installation Point 1 (right axis) and the data of SSMI/S microwave radiometer (left axis).

The passive microwave sensor SSMI/S (Special Sensor Microwave Imager/Sounder) onboard Satellite F17 of the DMSP (Defense Meteorological Satellite Program), was an improved version of SSM/I (Special Sensor Microwave/Imager). It acquires multiband microwave brightness temperature data, which are widely applied in estimations hydrological parameters, including soil moisture, sea ice, freezing, and melting [31,32]. Here, SSMIS microwave brightness temperature product, specifically the vertical polarization data of 91.7 GHz, in spatial resolution of 12.5 km and from July 2012 to July 2013, were compared to the time series of surface snow temperature measured by Installation Point 1 at 14:00 UTC. The comparison in Figure 9 showed that the high-frequency components of the two time series were obviously relevant. The low-frequency components had consistent trends in winter (from March to November) but were obviously different in summer (from December to March).

Microwave brightness temperature measures the microwave energy emitted by surface objects. It is both determined by the temperature and the emissivity of the surface objects. In Antarctica, because of freezing and melting in winter and summer, there were considerable changes in surface emissivity. The main effects on brightness temperature came from surface changes, especially in terms of the high-frequency component with lower penetrating power. The figure suggests that as the winter ended and the summer began, the surface temperature around Installation Point 1 increased and the liquid water content generally increased. This led to a sharp decrease in the surface emissivity (water has relatively low emissivity of approximately 0.5). In the winter, high-frequency microwaves can reflect the temperature changes on the surface in a relatively objective way. Figure 9 indicates that during the winter, high-frequency microwave data of 91.7 GHz generally reflected the temperature change; but, in the summer, with increased water, the mixing ratio of ice and water caused fluctuations and delays of the microwave signal. Part of the fluctuations in high-frequency microwave data may also arise from the change of atmospheric conditions.

### 5.3. Detection of Freezing-Melting Onset 

**Figure 10:**
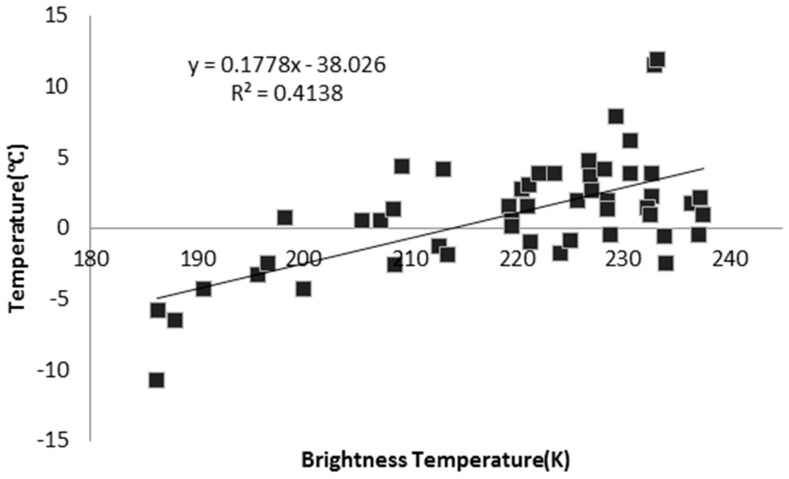
Detection of the freezing-melting onset.

The edge detection method is adopted here to detect the freezing/melting of the surface snow/ice based on wavelet transformation [33]. The method applies multi-scale wavelet transform to the time series of brightness temperature data in the 19 GHz horizontally polarized channel of the SSM/I passive microwave sensor, finds the edge of signal change, and determines the optimal threshold to locate the precise date of melting or freezing. Figure 10 presents the detected result of melting/freezing data near Zhongshan Station in Antarctica. Firstly, the brightness temperature of 19 GHz horizontally polarized channel (short as Tb19) is validated with ground measured snow surface temperature, and the result is shown as the scattered points in the right part of Figure 10. The observation frequency of surface temperature at the selected nodes (69°26′23′′ S, 76°16′56′′ E) was one for every 10 min. The validation data of ground temperature was the average of the five highest temperatures during the whole day. The time of validation lasted from 24 December 2010, to 11 February 2011. The left part of Figure 10 shows the freezing-melting date determined by the Tb19 time series with the wavelet transform analysis. The yellow line represents the date of melting onset, i.e., the 168th day of the year; and the green line represents the date of freezing onset, i.e., the 224th day of the year. The red curve is the Tb19 time series from 1 July 2010 to 30 June 2011; and the blue curve is the observation of snow surface temperature from the WSOOP node. In this example, the ground observations proved that the surface temperature rose above 0 °C during the dates between the detected melting onset and freezing onset, thus indirectly proving that monitoring melting/freezing status in the Antarctic with satellite remote sensing is applicable.

## 6. Conclusions

In these experiments, the field data from the WSOOP together with the ground observation data at Zhongshan Station Antarctica were used to validate the remote sensing products. The results of remote sensing retrieval were consistent with those of the ground observation; however, there were still some differences, which were mainly caused by three factors. The first factor was the errors of the remote sensing products [18]. The second factor was the inconsistent scales of the ground observation and remote sensing observation. Finally, the LST retrieved by remote sensing was the temperature of the snow surface, whereas the snow temperature observed by wireless sensors was at a certain depth [10]. Increasing the number of installation points could improve the accuracy of measurements in Antarctica. For the operations which have not been maintained for nearly two years, the WSOOP was able to acquire the first-hand ground observation data of the Antarctica. Through continuous monitoring of the platform’s performance, areas for improvement were also identified, such as reinforcing bidirectional communications, and the possibility of remote setting, managing, and monitoring the platform.

Although the remote sensing technology is largely affected by factors such as climate and satellite travel paths in acquiring temporally continuous observational data, it was and will still be an important method for scientific research in polar regions due to its capability in producing instantaneous images to cover vast areas. The experiments had shown the WSOOP system can acquire long-term, site-specific and continuous data, which is complementary to remote sensing data and can be used to validate the remote sensing products. This is informative and helpful in monitoring as well as improving the accuracy of remote sensing technology in polar region.

## Figures and Tables

**Table 1:** Sensor characteristics in the meteorological/snow instrument.

Name	Type	Accuracy	Size	Operating Temperature	Company
Temperature and Relative Humidity Probe	HMP45C	±2%RH/±0.2 °C	Length: 25.4 cm	0%–100%RH/−39.2 °C–+60 °C	Campbell Scientific, Inc. (Logan, UT, USA)
			Diameter: 2.5 cm		
AT Sonic Ranging Sensor	SR50A	±1 cm or 0.4% of distance to target	Length: 10.1 cm	−45 °C–+50 °C	Campbell Scientific, Inc. (Logan, UT, USA)
			Diameter: 7.6 cm		
Pyranometer	CMP11	<±2.5%	Width: 7.9 cm	−40 °C–+80 °C	Campbell Scientific, Inc. (Logan, UT, USA)
			Height: 6.7 cm		
			Dome Diameter: 3.2 cm		
Pressure Sensor	MS5534c	0.1 mbar	9 mm × 9 mm	−40 °C–+125 °C	Intersema (Bevaix, Switzerland)
Temperature Sensor	PT100	±0.010 °C	2 cm	−85 °C–+85 °C	Anhui Jingshifangyuan Inc. (ChiZhou, China)

**Table 2:** The installation location of the wireless sensor network devices.

Name	Location	Factors
Primary Node	69°28′45.85′′ S, 76°20′39.40′′ E	Air temperature, air humidity, wind speed, wind destination, snow depth, atmospheric pressure and nine-tier snow temperature (the temperature in the first layer is named the surface snow temperature)
Child Node	69°26′1′′ S, 76°16′55′′ E	Air temperature, air humidity, and nine-tier snow temperature
Mountpoint1	69°31′56.38′′ S, 76°14′45.10′′ E	Air temperature, air humidity, and nine-tier snow temperature
Mountpoint2	69°36′11.45′′ S, 76°13′22.75′′ E	Air temperature, air humidity, and nine-tier snow temperature

## References

[1] Li B.R., Liu S.L., Guo J.X., Xi Y. Polar remote sensing field validation system and comprehensive experiment. Proceedings of the 26th Annual Meeting Chinese Geophysical Society.

[2] Chen L.Q. (2002). Study on the role of the Arctic and Antarctic regions in global change. Earth Sci. Front..

[3] Giammarini M., Isidori D., Pieralisi M., Cristalli C., Fioravanti M., Concettoni E. (2016). Design of a low cost and high performance wireless sensor network for structural health monitoring. Microsyst. Technol..

[4] Silvani X., Morandini F., Innocenti E., Peres S. (2015). Evaluation of a Wireless Sensor Network with Low Cost and Low Energy Consumption for Fire Detection and Monitoring. Fire Technol..

[5] Gui Y., Tao Z.G., Wang C.J., Xie X. (2011). Study on remote monitoring system for landslide hazard based on wireless sensor network and its application. J. Coal Sci. Eng. China.

[6] López J.A., Garcia-Sanchez A.J., Soto F., Iborra A., Garcia-Sanchez F., Garcia-Haro J. (2011). Design and validation of a wireless sensor network architecture for precision horticulture applications. Precision Agric..

[7] Gong P. (2007). Wireless Sensor Network as a New Ground Remote Sensing Technology for Environmental Monitoring. J. Remote Sens..

[8] Gong P., Cheng X., Li X.H., Wang L., Shen S.Q. (2009). The Application of Wireless Sensor Network Technology in Ground Environment Sensing. J. Remote Sens..

[9] Liu T., Muye N.I.U., Wang Z., Huang X., Cao L., Tian Z. (2015). An Effective Antarctic Ice Surface Temperature Retrieval Method for MODIS. Photog. Eng. Remote Sens..

[10] Wang Y., Hou S. (2011). Spatial distribution of 10 m firn temperature in the Antarctic ice sheet. Sci. China Earth Sci..

[11] Heavner M., Fatland R., Hood E., Connor C., Habermann M., Gerner L. (2008). Monitoring Lemon Glacier Using a Wireless Sensor Network in Juneau. Joint Meet. Geol. Soc. Am..

[12] Roberta P. (2004). Surface albedo measurements over Antarctic sites in summer. J. Geophys. Res. Atmos..

[13] Broeke M.V.D., As D.V., Reijmer C., Wal R.V.D. (2004). Assessing and improving the quality of unattended radiation observations in Antarctica. J. Atmos. Ocean. Technol..

[14] Li X.H., Cheng X., Yang R.J., Zhang H.J., Zhang J.L., Hui F.M., Wang F. (2014). A multi-interface ice and snow remote monitoring platform in the Polar region. IEEE Sens..

[15] Hugues G., Braida M., Crosta X., Mairesse A., Masson-Delmotte V., Mathiot P., Neukom R., Oerter H., Philippon G., Renssen H. (2012). Antarctic temperature changes during the last millennium: Evaluation of simulations and reconstructions. Quat. Sci. Rev..

[16] Marshall G.J., Di Battista S., Naik S.S., Thamban M. (2011). Analysis of a regional change in the sign of the SAM-Temperature relationship in Antarctica. Clim. Dyn..

[17] Bian L., Lin Z., Zhang D., Zheng X., Lu L. (2012). The vertical structure and seasonal changes of atmosphere ozone and temperature at Zhongshan Station over East Antarctica. Sci. China Earth Sci..

[18] Xin Y., Bian L., Rinke A., Dethloff K. (2014). Simulation and evaluation of 2-m temperature over Antarctica in polar regional climate model. Sci. China Earth Sci..

[19] Liston G., Winther J. (2005). Antarctic Surface and Subsurface Snow and Ice Melt Fluxes. J. Clim..

[20] Qiu Y.B., Guo H.D., Shi J.C., Kang S.C., James R.W., Juha L., Jiang L.M. The preliminary analysis of snow monitoring using AMSR-E and winter snow campaign over Tibet platean, China. Proceedings of 2010 IEEE International Geoscience & Remote Sensing Symposium.

[21] Juha L., Jouni P., Andrew R., Anna K., Qiu Y.B., Chris D. (2010). Multiple Layer Adaptation of HUT Snow Emission Model: Comparison with Experimental Data. IEEE Trans. Geosci. Remote Sens..

[22] Qiu Y., Guo H., Shi J., Lemmetyinen J. An emissivity-based land surface temperature retrieval algorithm using AMSR-E microwave measurement. Proceedings of the IGARSS.

[23] Markus T., Stroeve J.C., Miller J. (2009). Recent changes in arctic sea ice melt onset, freezeup, and melt season length. J. Geophys. Res..

[24] Drobot S.D., Anderson M.R. (2001). An improved method for determining snowmelt onset dates over Arctic sea ice using scanning multichannel microwave radiometer and special sensor microwave/imager data. J. Geophys. Res..

[25] Smith D.M. (1998). Observation of perennial Arctic sea ice melt and freeze-up using passive microwave data. J. Geophys. Res..

[26] Bliss A.C., Anderson M.R. (2014). Daily Area of Snow Melt Onset on Arctic Sea Ice from Passive Microwave Satellite Observations 1979–2012. Remote Sens..

[27] Torinesi O., Fily M., Genthon C. (2003). Variability and trends of the summer melt period of Antarctic Ice margins since 1980 from Microware sensors. J. Clim..

[28] Wan Z., Li Z.-L. (1997). A Physics-Based Algorithm for Retrieving Land-Surface Emissivity and Temperature from EOS/MODIS Data. IEEE Trans. Geosci. Remote Sens..

[29] Wan Z., Zhang Y., Zhang Q., Li Z.-L. (2002). Validation of the land-surface temperature products retrieved from Terra Mode rate Resolution Imaging Spectroradiometer data. Remote Sens. Environ..

[30] Wan Z., Li Z.-L. (2008). Radiance-based validation of the V5 MODIS land-surface temperature product. Int. J. Remote Sens..

[31] Abdalati W., Steffen K., Otto C., Jezek K.C. (1995). Comparison of Brightness Temperatures from SSM/I Instruments on the DMSP F8 and F11 Satellites for Antarctica and the Greenland Ice Sheet. Int. J. Remote Sens..

[32] Comiso J.C. (1986). Characteristics of Arctic Winter Sea Ice from Satellite Multispectral Microwave Observations. J. Geophys. Res..

[33] Liu H.W., Wang L., Jezek K. (2005). Wavelet-Transform based edge detection approach to derivation of snowmelt onset, end and duration from satellite passive microwave measurements. Int. J. Remote Sens..

